# Injection of the Broad Ligaments for Prolapsus Uteri.—II

**Published:** 1911-08-05

**Authors:** J. Inglis Parsons

**Affiliations:** Surgeon to the Chelsea Hospital for Women.


### Hospital Clinics.

### INJECTION OF THE BROAD LIGAMENTS FOR PROLAPSUS UTERI.—II.

By J. INGLIS PARSONS, M.D., M.R.C.P., M.R.C.S., Surgeon to the Chelsea Hospital for Women.

The difference in the amount of effusion produced by the injection in patients varies very widely. Sometimes one injection will hold up the uterus in a case of chronic procidentia and she may be allowed to go out in three weeks without even repairing the perineum or wearing a pessary; while at the other end of the scale, the effusion may be so weak that a second is necessary to produce satisfactory results. But whatever the amount of effusion produced, it is necessary to bear in mind that it is not thoroughly organised into strong fibrous tissue under six months. This is only what we should expect from our knowledge of the repair that goes on round an ordinary joint after dislocation.

The general surgeons have often had to point out, in the case of the shoulder joint, that if a great strain is thrown on it too soon after reduction the dislocation will recur. The same laws of nature are just as inexorable when applied to the uterus, and the prolapse is certain to recur if too great a strain is thrown on the ligaments while the repairing lymph is still soft and unable to bear it; although the same ligament at a later date might be quite able to stand any strain. From the foregoing it can be understood how necessary it is to form an adequate estimate of the amount of effusion, and also to consider this in conjunction with the duration and extent of the prolapse, before coming to any decision as to how soon the patient can get up and go about.

It is by no means easy to feel how much lymph is effused, because the uterus is often held so high up after operation that the utero-pelvic band is almost out of reach.

If before operation a suitable pessary kept the uterus up properly, the patient in the first stage of prolapse may quite well get up and even go about a little in ten days' time, provided the pessary is re-inserted so as to take the strain off the newly-effused lymph. At the end of four to six months the pessary can be done away with, and the prolapse gives no further trouble.

### Points in Technique of Operation.

Further experience has led me to modify some points in the technique of the operation. With regard to the quantity of quinine injected, at first I did not give more than 30 or 40 minims of the solution on each side. This was not enough for old-standing cases and the injection had to be repeated. Therefore, the quantity has been gradually increased, until as much as 80 minims is injected on the patient's right side and 70 minims on her left. The reason for reducing the quantity on the left is that the space for injection is smaller on that side on account of the rectum.

The position of the bladder, and with it the ureters, has to be carefully considered when operating on cases of chronic procidentia. In some of these the uterus, in coming down, leaves the bladder behind; in others, it drags the bladder with it. In the latter circumstances, the ureters and the bladder walls become stretched, and when the uterus is replaced it will be found that the bladder comes down much lower than usual on each side. To obviate the possibility of puncturing the base of the bladder, I have several sizes of retractors for holding up the anterior vaginal wall. The longest and widest that can be passed should always be used. The assistant holding it should be directed to tilt the end up near the uterus, so as to pull the bladder well up out of the path of the needle. It is also advisable with this class of case to puncture the vaginal walls at a somewhat lower point.

The solution of quinine should be freshly made, because it seems to undergo a change after being kept. In cold weather the quinine in a few days will precipitate and form crystals. If the solution (12 grains of the sulphate of quinine in 30 minims of distilled water and 30 minims of dilute sulphuric acid) is not made up to the proper strength, it is needless to say that the operation will probably fail.

A fairly good and simple test is to have a standard solution in 1 in 20 carbolic. If a few drops of the solution of quinine are added to this a very dense white cloud is produced, and the eye soon becomes accustomed to the appearance produced by a standard solution. The small quantity of carbolic used can also serve to sterilise the needle used for the injection.

A platino-iridium needle is better than steel, because the latter becomes corroded by the acid and may break off in the tissues and give great trouble in its extraction.

There should be no rise of temperature after the injection. When it does occur it is generally at the end of five to seven days after operation. In two cases, who were none the worse for the experience, I found that the temperature was due to an invasion of the effusion by the bacillus coli from the rectum. In order to obviate this, the bowel before operation for two or three days is washed out with chinosol 1 in 4000, after the usual clearing out with an enema. For normal healthy patients this is not necessary, but it should always be done when there is much constipation or a general unhealthy condition of the system. The bowels after the operation must be kept open for the same reason, but purging should be avoided, because it tends to reduce the amount of lymph. For the first week after the operation the same wash-out with chinosol after the bowels have acted is advisable in doubtful cases.

Another important point is to instruct the patient after the operation to lie on her face as much as possible. This throws the uterus forward into a position of anteversion. When the patient gets up there is less strain on the new ligaments if the uterus is anteverted.

There appears to be little or no risk from this operation and I have not lost any patient from it.

On the other hand, it must be done with proper care and the precautions already indicated. It would be quite easy for any careless operator to run the needle into the ureter, bladder, or uterine artery. I have now done over 200 cases, and in only three of these was there any inflammation or parametritis. All three patients got quite well and the prolapse was cured.

Taking the cases of prolapse in the first stage, nearly every case is cured provided the after-treatment is carried out properly. Of those in the second stage quite 90 per cent. recover. Even with patients in the third stage of procidentia, the uterus keeps up after the operation in 75 per cent. I had an opportunity recently of examining, with Dr. Aikman, a patient who had procidentia and was operated on nine years ago. Although the uterus was heavier than usual from the development of a fibroid since the operation, there was no prolapse whatever and the new ligament could be distinctly felt on each side.

Some eight years ago Dr. Cowen, of New Malden, Surrey, sent me a case of chronic procidentia, and since then a number of other cases. Writing to me this week, April 14, 1911, he states in his letter: "You have injected a good number of my patients for prolapse of the uterus, and all that have remained in this neighbourhood are still blessing you, and when I can hear of those that have removed the answer is still 'all right.'"

Perhaps the best criterion of the success of an operation is not the results obtained by the inventor of it, but the results of those who have followed in his footsteps. I therefore quote the following:—

Dr. J. Aikman, Guernsey, has operated on seven cases, all successful. The first was done in 1900.

Dr. Rice, of Derby, thirty-one patients. All but one of these was successful. Three of the patients had children after the operation without any return of the prolapse. One patient was aged seventy-two.

Dr. Lea Wilson, Nasik, India, operated on twelve patients. Ten of these were successful, and one was seven months pregnant.

Dr. Eugene Carlier, of Brussels, operated on fifteen cases and was very satisfied with the results.

Dr. S. Kent, of Bexhill, has operated on nine patients, all successful. Two of these had children without recurrence of the procidentia.

Dr. Crewdon Thomas published a case in the *British Medical Journal* of a successful result, followed by conception and parturition, with no return of the prolapse.

Dr. Eden in one case of procidentia succeeded in curing the patient after ventro-fixation had failed.

Another morbid condition for which this operation may prove of some value is prolapse of the rectum in women. The first case, done two years ago for Dr. Cowen, has proved very successful. As the rectum lies just behind the utero-pelvic band on the left side, it is only necessary to inject rather lower to form a support for the bowel as well as the uterus.

